# The Cell Adhesion and Proliferation Enhancement Impact of Low-Temperature Atmospheric Pressure Plasma-Polymerized Heptylamine on the Surface of Ti6Al4V Alloy

**DOI:** 10.3390/ma16196450

**Published:** 2023-09-28

**Authors:** Di Le, Jie Pan, Haixia Xing

**Affiliations:** Department of General Dentistry, Peking University School and Hospital of Stomatology & National Center for Stomatology & National Clinical Research Center for Oral Diseases & National Engineering Research Center of Oral Biomaterials and Digital Medical Devices, Beijing 100081, China; ledi@pkuss.bjmu.edu.cn (D.L.); pan-jie@bjmu.edu.cn (J.P.)

**Keywords:** surface modification, heptylamine, plasma polymers, polymerization, titanium alloy, Ti6Al4V

## Abstract

To chemically functionalize the Ti6Al4V alloy surface, a custom-made low-temperature atmospheric pressure plasma reactor device was used to polymerize heptylamine on it. The effect of different deposition times, an important process parameter, was also investigated. For each deposition time group, the surface morphology was observed via scanning electron microscopy (SEM). The surface chemical content was analyzed via X-ray photoelectron spectroscopy, and surface hydrophilicity was measured via water contact angle. The adhesion of bone marrow stromal cells (BMSCs) on the modified Ti6Al4V alloy surfaces was also observed via SEM. A quantitative evaluation of cell proliferation was performed via the Cell Counting Kit-8 assay. The results revealed that amino groups were introduced on the Ti6Al4V alloy surface via plasma-polymerized heptylamine (PPHA). The percentages of NH_2_/C for various deposition times (0 s, 30 s, 45 s, 60 s, 90 s, and 120 s) were 3.39%, 5.14%, 6.71%, 6.72%, 7.31%, and 7.65%. A 30 s, 45 s, and 60 s deposition time could significantly increase surface hydrophilicity with a mean water contact angle of 62.1 ± 1.6°, 65.7 ± 1.1°, and 88.2 ± 1.4°, respectively. Meanwhile, a 60 s, 90 s, and 120 s deposition time promoted BMSCs cell adhesion and proliferation. However, this promotion effect differed non-significantly among the three groups. In conclusion, the introduction of amino groups on the Ti6Al4V alloy surface exhibited surface modification and enhancement of cell adhesion and proliferation, which was partially associated with deposition time.

## 1. Introduction

Biomedical applications for titanium and its alloys include the production of artificial joints, dental implants, cardiovascular implants, and fracture repair fixation devices. Ti6Al4V alloy accounts for more than 50% of all alloy applications because of its specific strength, corrosion resistance, and biocompatibility [1]. The biocompatibility of Ti6Al4V alloys was better compared to that of traditional alloys such as Co-Cr-based alloy or steel but performed poorly compared to the new generation of beta titanium alloys, mainly due to the presence of Al and V content. Decreased attachment of the bone to Ti6Al4V alloy implants was observed compared to that of commercially pure titanium [2]. The bone growth on an implant surface is influenced by the activity of differentiated bone stromal cells. Ti6Al4V alloy exhibits a relative hydrophobicity [3] that could potentially hinder the adhesion and spread of bone marrow stromal cells (BMSCs). Consequently, a range of surface modification techniques have been employed on Ti6Al4V alloy to achieve distinct physical morphology and chemical composition, influencing surface properties such as roughness, hydrophilicity, biocompatibility, and bioactivity.

Plasma surface modification techniques that do not affect the material bulk properties have garnered significant attention in recent years. Plasma polymerization has emerged as a highly effective technique for depositing ultrathin nanocoating on a variety of surfaces. Polymeric coating on metal can form a protective, decorative, and functional coating. The chemical properties of the original substrate surface were partially or completely replaced by polymeric coating, while the bulk material’s mechanical properties were retained [4]. Plasma polymerization on the Ti6Al4V alloy has exhibited the potential to improve cell adhesion and bone growth, with additional benefits derived from the nanoscale plasma polymer film [4,5,6]. In plasma polymerization, an organic monomer is introduced into the reactor chamber under rough (or low) vacuum conditions. Next, a microwave or radiofrequency (RF) generator creates an electrical field between electrodes embedded in the chamber. When subjected to an electrical field, organic monomers undergo ionization, resulting in the formation of free radicals, charged molecules, ions, and electrons. Subsequently, these species condense and polymerize on the specific substrate, which is precisely positioned on the electrodes [7]. Plasma polymerization exhibits a strong dependence on the specific system employed, wherein the outcomes are contingent upon the reactor’s conditions and operational procedures. Apart from the pressure within the reactor chamber, the typical parameters of one reactor system encompass discharge wattage, deposition time, the organic monomer employed, monomer flow rate [7,8], the temperature at the substrate position, etc. The surface properties of thin polymer films resulting from plasma polymerization are influenced by these parameters.

Plasma polymerization typically operates at 50 mTorr system pressure (6.67 Pa) and a discharge wattage of 5 to 10 Watt, 1 sccm (standard cubic centimeter of gas per minute) monomer flow rate [7]. As described in previous research, the reactor chamber is normally evacuated to a rough (or low) vacuum [9,10,11,12]. The vacuum equipment must be configured in a complicated and expensive manner. Implosion is a risk in high-vacuum conditions, so it is important to avoid contact with the glass chamber and to provide a protective barrier to prevent this [8]. Utilizing atmospheric pressure plasma treatment, which eliminates the need for vacuum equipment, could avoid implosion, decrease manipulation difficulties, and save time and cost [13]. In light of this, our research group has successfully designed and constructed an atmospheric pressure plasma reactor while also fine-tuning various device parameters for optimal performance.

Discharge wattage and deposition time are important plasma reactor parameters. Plasma polymer thickness and chemical content were influenced predominantly by the glow discharge power and deposition time. These two operational conditions varied widely in previous research. The discharge wattage was reported from 5–10 W [7,10,11] up to 50 W [12]; the deposition times ranged from 30 s, 60 s [8], 90 s [11,12], 6 min [14] to as high as 20 min [9,10] in various experiments [1,8,15]. Amino groups are among the most prevalent functional groups in biological systems. Amine-functionalized surfaces achieved through plasma polymerization have been proven to boost cell attachment and enhance cell migration and intracellular signaling in vital cells [4,16]. The different monomer vapors with amino groups included allylamine [5,15], n-heptylamine [8,10,11,12], etc. Previous research has indicated that n-heptylamine is less toxic and has a more stable monomer pressure compared with allylamine [17]; it also exhibits an adequate presence of amino functional groups suitable for biological applications [17]. Plasma-polymerized heptylamine (PPHA) exhibits osteoinductive and osteogenic potential, demonstrating a significant increase in alkaline phosphatase (ALP) activity [11], along with enhancement of adhesion behavior of human bone cells [5] and of bone growth in animal testing [5]. In the present investigation, a bespoke plasma reactor without vacuum equipment was constructed to generate surfaces featuring primary amines via the utilization of n-heptylamine monomer. The plasma polymerization process was conducted in atmospheric conditions. This plasma reactor exhibits several advantages, including cost-effectiveness, absence of vapors, expedited deposition, and enhanced efficacy.

This study utilized heptylamine plasmas to fabricate thin polymer films incorporating amino groups, which were subsequently polymerized on the surface of Ti6Al4V alloy. The investigation focused on the impact of varying PPHA deposition times on the morphology, chemical composition, water contact angle, adhesion, and BMSCs’ proliferation. The hypothesis posited that the utilization of PPHA film could potentially augment the bioactivity of Ti6Al4V alloy.

## 2. Materials and Methods

### 2.1. Description of the Custom-Made Low-Temperature Atmospheric Pressure Plasma Reactor System

**Figure 1:**
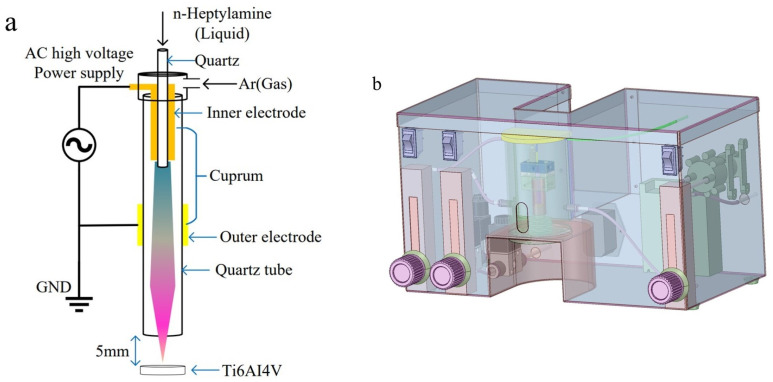
Low-temperature atmospheric pressure plasma reactor device. (**a**) Schematic diagram of device and plasma polymerization process. GND—gross national demand. AC—alternating current. (**b**) Design diagram of the device.

Plasma polymerization was conducted using a plasma reactor specifically designed for this purpose. Figure 1 contains a schematic representation of the reactor, which consists of multiple sub-units. The alternating current (AC) high-voltage generator operated at a consistent frequency of 30 kHz. Throughout the plasma polymerization process, the pressure within the reactor chamber was maintained at atmospheric levels, while the temperature within the reactor chamber was approximately 20 °C.

The central component of the reaction polymerization chamber consisted of a cylindrical quartz glass tube of 8 cm in length, 1 cm in diameter, and 1 mm in wall thickness. Positioned at the inlet side of the quartz tube, the internal horizontal copper electrode took the form of a hollow cylinder with a diameter of 0.5 cm, serving as the connection to the high-voltage positive electrode. Surrounding the outer wall of the quartz glass tube, the outer copper electrode, with a diameter of 1 cm, was wound and linked to the high-voltage negative electrode. The outer copper electrode was positioned approximately 2 cm below the inner electrode, creating a Dielectric Barrier Discharge (DBD) structure. The plasma was generated within this 2 cm discharge gap. The samples were positioned at 5 mm beneath the quartz glass tube.

### 2.2. Preparation of Ti6Al4V Alloy Disks and Plasma Polymerization

The Ti-6Al-4V alloy disks (10 mm in diameter and 2 mm high) were manufactured via selective laser melting. The initial Ti6Al4V powder used in the fabrication process was purchased from the manufacturer (Optimal Material Technology Co., LTD, Chengdu, China). The chemical composition of virgin Ti6Al4V powder is Ti 89.35 wt%, Al 6.29 wt%, and V 4.04 wt%. In total, 10% of particle distribution occurred from 15 to 25 µm, while 90% of particles occurred from 45 to 53 µm. The Ti6Al4V alloy disks were manufactured using the M2 Concept Laser Cusing (Concept Laser, KOCAELİ, Lichtenfels, Germany). The machine is equipped with a 200 W (cw) fiber laser. Argon was used as a protective atmosphere during the process, and the O_2_ content was maintained below 0.05%. The procedure was processed with a setting power of 200 W, a scanning speed of 7 mm/s, a focus diameter of 50–500 μm, and a single-layer thickness of 20–80 μm. Before each experiment, the powder humidity was less than 10%. Following fabrication, all disks underwent a series of polishing steps at magnifications of 80×, 300×, 400×, 800×, 1000×, 2000× and 4000×. Subsequently, the disks were subjected to ultrasonic cleaning with acetone, 99% ethanol, and demineralized water for 15 min each.

The three-step procedure for plasma polymerization commenced with the placement of the cleaned Ti6Al4V disks onto the platform. To eliminate air from the reactor chamber and prevent oxygen from interfering with the polymerization process, the chamber was filled with nitrogen at a gas flow rate of 100 mL/min. Refrigeration equipment was installed in the device, and the temperature was set to 20 °C. When the nitrogen left the cylinder and flowed through the refrigeration equipment, the temperature of the nitrogen was reduced to 20 °C. Subsequently, the cleaned Ti6Al4V alloy disks underwent decontamination and activation through exposure to a continuous-wave argon plasma (9 W, atmospheric pressures) for 5 s. Next, liquid reagent n-heptylamine (HA, 99% purity, Aladdin, Shanghai, China) was introduced into the quartz tube via PVC tubing (2 mm internal diameter; 4 mm external diameter) from a container. To ensure precise regulation of the liquid flow, a spiral micrometer was employed manually. For each Ti6Al4V disk, the spiral micrometer was rotated half a turn, resulting in the dispensation of 7.06 μL of n-heptylamine liquid. The n-heptylamine plasma was activated at a voltage of 30 V and a current of 0.3 A (equivalent to 9 W) while being carried by argon plasma. The plasma reached lengths of up to 4 cm before being expelled through the outlet of a quartz glass tube and polymerized on the surface of Ti6Al4V disks. After varying deposition times of 30 s, 45 s, 60 s, 90 s and 120 s, the generator was switched off to stop the plasma. To optimize plasma polymerization, the samples were then left in the chamber for an additional 30 s. Prior to surface analysis and cell culture, samples were stored in a sealed vacuum container to avoid air infiltration.

### 2.3. The Surface Morphology of Ti6Al4V Disks Subsequent to n-Heptylamine Plasma Polymers Polymerization

The morphology of the PPHA-Ti6Al4V alloy was observed via scanning electron microscopy (SEM) (Gemini 300, Carl Zeiss, Oberkochen, Germany). The samples were coated with gold using a sputter coater prior to observation. The surface morphology images of the central treatment area were captured using an accelerating voltage of 1 kV and a magnification of 1000×.

### 2.4. X-ray Photoelectron Spectroscopy (XPS)

The chemical composition of PPHA on Ti6Al4V disks at various deposition times (0 s, 30 s, 45 s, 60 s, 90 s, and 120 s) was identified by using the Thermo Scientific K-Alpha+ XPS system (ThermoFisher, Thermo Fisher Scientific Inc., Hillsboro, OR, USA). The XPS system, consisting of a monochromatic Al-Kα source (hv = 1486.6 eV), was operated at a power of 15 mA, 15 kV. XPS was used to detect specific electron energies and identify chemical bonds based on peak shifts, excluding hydrogen detection, in a vacuum chamber (5 × 10^−9^ mbar). Survey spectra were recorded and analyzed to evaluate the atomic percentages of carbon, oxygen, and nitrogen in the thin PPHA film. The binding energy scale of the C1s peak was adjusted to 284.8 eV to account for the C–C/C–H component. All peaks were fitted with reference to 284.8 eV using XPSPEAK41 software 4.1.0.0. The determination of peak intensities was performed using Shirley-background subtraction. The XPS fitting curves of different samples were processed using OriginPro 2023 learning edition (OriginLab Corporation, Northampton, MA, USA). Subsequently, the relative chemical contents (C%, N%, O%, NH_2_/C%) were calculated by employing peak area normalization.

### 2.5. Water Contact Angle Measurement

Water contact angles were measured using the sessile drop method (JY-82B Kruss DSA, Hamburg, Germany) at 25 °C to evaluate the hydrophilicity of the PPHA-Ti6Al4V alloy with deposition times of 0 s, 30 s, 45 s, 60 s, 90 s, and 120 s. Approximately 5 µL of distilled water was dropped onto each sample surface, and images of the water droplet were captured at 5 s intervals using a high-resolution camera. This procedure was repeated three times. The contact angles were calculated based on the acquired images.

### 2.6. Cell Culture and Seeding

Murine bone marrow-derived mesenchymal stem cells (BMSCs) were purchased from Cyagen Biosciences, Inc., Guangzhou, China. The culture medium contained murine MSC basal medium (Cyagen Biosciences, Inc., Guangzhou, China) supplemented with 10% MSC-qualified fetal bovine serum (FBS, Gibco, Waltham, MA, USA), 100 IU/mL penicillin–streptomycin, and 2 mM glutamine (all from Cyagen Biosciences, Inc., Guangzhou, China). The medium was refreshed every 2 to 3 days. After reaching 80–90% confluence, cells were detached using 0.25% trypsin/EDTA (Gibco, Life Technologies, Waltham, MA, USA) and subcultured at a density of 5 × 10^5^ cells in a T25 flask. The BMSCs were passaged three times before seeding. Prior to cell seeding, PPHA-Ti6Al4V alloys with different deposition times were ultrasonicated subsequently in acetone, 99% ethanol, and demineralized water for 15 min each. After that, the samples were sterilized with ultraviolet (UV) light for 30 min and immersed in phosphate-buffered saline (PBS) for 30 min. For cell seeding, 1 × 10^4^ BMSCs were added into a 24-well plate containing 800 μL of fresh culture medium and then incubated in 37 °C, 5% CO_2_ incubator. The experimental samples were categorized based on the duration of PPHA deposition times, namely 0 s, 30 s, 45 s, 60 s, 90 s, 120 s, and the control group. The culture medium was replenished every 2–3 days.

### 2.7. Cellular Adhesion Morphology Observation

The cellular morphology of BMSCs was assessed 6 h after seeding on PPHA-Ti6Al4V alloy disks using a Tescan MIRA LMS field-emission scanning electron microscope (SEM, Tescan Ltd., Brno, Czech) with an accelerating voltage of 15 keV. Prior to imaging, the BMSCs were washed with phosphate-buffered saline (PBS) and fixed with 4% paraformaldehyde for 30 min. The samples underwent a triple rinsing process using deionized water, followed by dehydration using a sequence of graded alcohol solutions at concentrations of 30%, 50%, 70%, 80%, and 90% for 15 min each, and 100% twice for 15 min, before being left to air-dry overnight. For better visualization, cells on Ti6Al4V disks were desiccated and coated with gold via spray application prior to SEM characterization.

### 2.8. Cell Proliferation on PPHA-Ti6Al4V Alloy Disk

Cell proliferation was assessed in various deposition time groups using a Cell Counting Kit-8 assay (CCK-8, Dojindo Molecular Technology, Kumamoto, Japan), which relies on the utilization of a water-soluble tetrazolium salt (WST-8). The Ti6Al4V alloy disks were placed within wells of 24-well plates with BMCs cell density of 2 × 10^4^ cells/mL. The cells were co-cultured with the PPHA-Ti6Al4V alloy disks for varying durations of 1, 3, 5, and 7 days at 37 °C in a 5% CO_2_ incubator. At each time point, samples were washed three times with PBS. Then, 450 µL of serum-free medium and 50 µL of Cell Counting Kit-8 solution mixed with WST-8 reagent were added to each well. The plate was then incubated for an additional 1 h under light protection. Subsequently, 100 µL supernatant from each well was pipetted to a 96-well plate, and the absorbance (OD values) was measured at 450 nm using a microplate reader (Bio-Rad 680; Microplate Master, Totowa, NJ, USA) in accordance with the manufacturer’s instructions. Three replicate wells were established for each deposition time group.

### 2.9. Statistical Analysis

All experimental results from water contact angle and CCK8 assay were expressed as the mean ± standard deviation (SD). These results of the six groups were analyzed with one- or two-way Analysis of Variance (ANOVA) using SPSS Statistics 24.0 (IBM, Armonk, NY, USA) for intergroup comparisons. Meanwhile, Tukey’s test was used for pairwise comparisons between multiple groups. *p*-values less than 0.05 were considered statistically significant.

## 3. Results

### 3.1. The Surface Morphology of PPHA-Ti6Al4V Alloy via SEM

**Figure 2:**
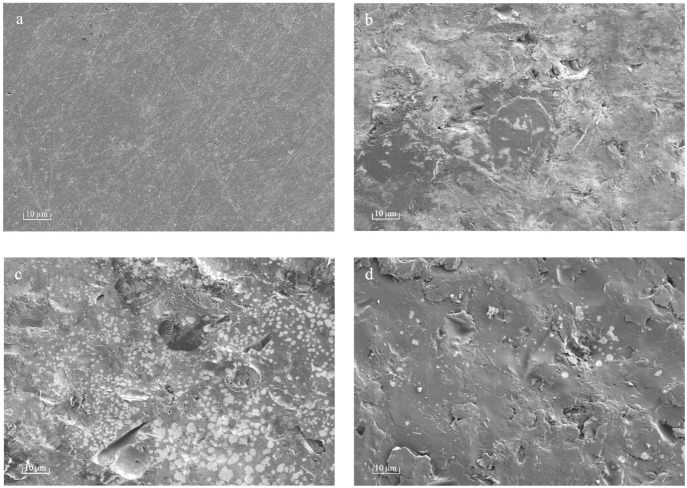

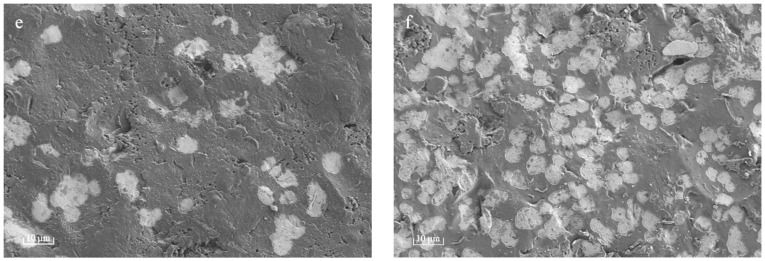
SEM images at 1000× magnification. (**b**–**f**) illustrate the presence of the PPHA on Ti6Al4V alloy surface. (**a**) Deposition time 0 s; (**b**) deposition time 30 s; (**c**) deposition time 45 s; (**d**) deposition time 60 s; (**e**) deposition time 90 s; (**f**) deposition time 120 s.

Figure 2 shows a series of SEM micrographs representing the surface morphology of the PPHA-Ti6Al4V alloy. This depicts the formation of PPHA on the Ti6Al4V alloy surface after plasma polymerization, as expected. The Ti6Al4V alloy presents a flat and smooth surface (Figure 2a). Upon 30 s deposition, the PPHA polymer polymerized on a Ti6Al4V alloy surface (black region), resulting in a relatively rough surface (Figure 2b). Subsequently, as the deposition time extended to 45 s and 60 s, a noticeable increase in the relative area of polymerized polymer occurred (Figure 2c,d). However, when the deposition time was further prolonged to 90 s and 120 s, a significant portion of the polymer was stripped away (white region) during the polymerization process (Figure 2e,f).

### 3.2. XPS Survey Spectra of PPHA-Ti6Al4V Alloy Surface

**Figure 3:**
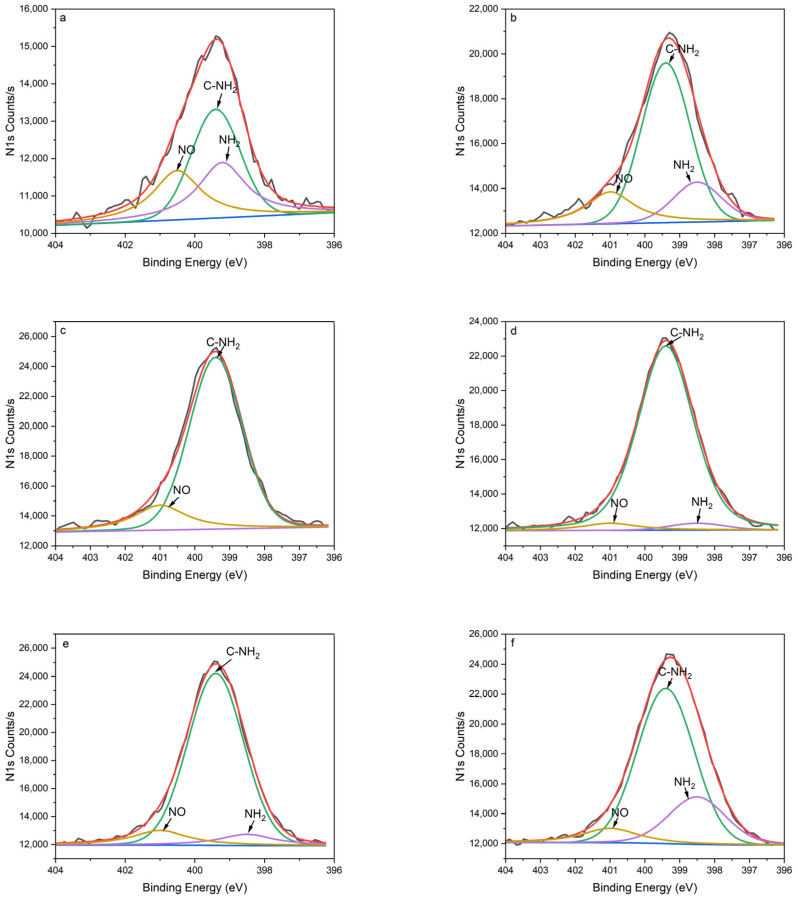
XPS N1s spectra of PPHA-Ti6Al4V alloy for various deposition times: (**a**) 0 s; (**b**) 30 s; (**c**) 45 s; (**d**) 60 s; (**e**) 90 s; (**f**) 120 s. The energy level was as follows: NH_2_: ~398.5 eV, purple line; C-NH_2_: ~399.4 eV, green line; NO: ~401.0 eV, orange line.

The plasma polymer was verified and characterized using X-ray photoelectron spectroscopy (XPS). The relative chemical composition and atomic ratios of PPHA are presented in Table 1, including C%, N%, O%, N/C%, NH_2_/C%, and NH_2_/N%. The percentage of NH_2_/C for various deposition times of 0 s, 30 s, 45 s, 60 s, 90 s, and 120 s were 3.39%, 5.14%, 6.71%, 6.72%, 7.31%, 7.65%, respectively, which indicated that the amino group was introduced onto the Ti6Al4V surface. As the deposition time increased, there was a gradual increase in the nitrogen content and NH_2_/C%. PPHA films formed within 120 s of deposition time still exhibited a significant presence of nitrogen functional groups on the Ti6Al4V surface. Furthermore, as presented in Figure 3, from peak fitting the N1s spectra, 72–95% of the total area (nitrogen) may be attributed to the amino group (-C–NH_2_, ~399.4 eV and -NH_2_, ~398.5 eV).

### 3.3. The Hydrophilicity of PPHA-Ti6Al4V Alloy Surface

**Figure 4:**
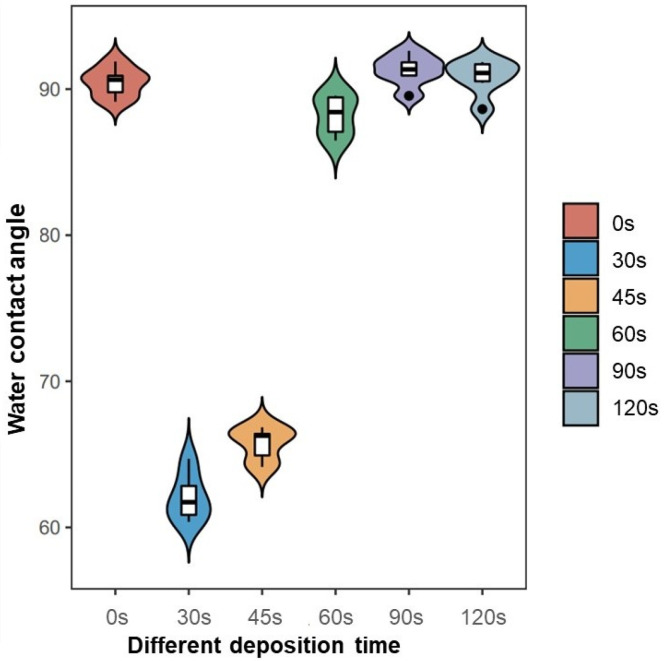
Influence of PPHA deposition times on water contact angle of Ti6Al4V alloy.

The hydrophilicity of the PPHA-Ti6Al4V alloy surface was examined by utilizing static water contact angle measurements. As presented in Figure 4, the water contact angle on the Ti6Al4V surface was 90.5° ± 1.0°. When the plasma deposition time increased to 30 s and, subsequently, to 45 s, the water contact angle significantly decreased to 62.1° ± 1.6°, 65.7° ± 1.1° (*p* = 0.0033 < 0.05, *p* = 0.0033 < 0.05). However, the water contact angle at a 60 s deposition time was 88.2° ± 1.4°, which was significantly smaller than the angle at 0 s (*p* = 0.016 < 0.05), but still significantly larger than angles at 30 s and 45 s (*p* = 0.0033 < 0.05, *p* = 0.0033 < 0.05). There was no significant difference among angles at deposition times of 0 s, 90 s, and 120 s (*p* > 0.05).

### 3.4. The Morphology Observation of BMSCs after Adhesion on PPHA-Ti6Al4V Surface

**Figure 5:**
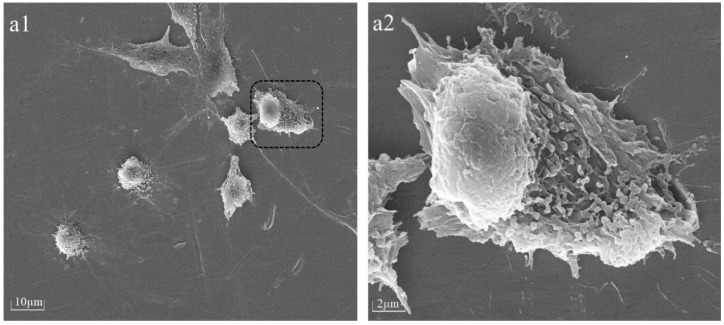

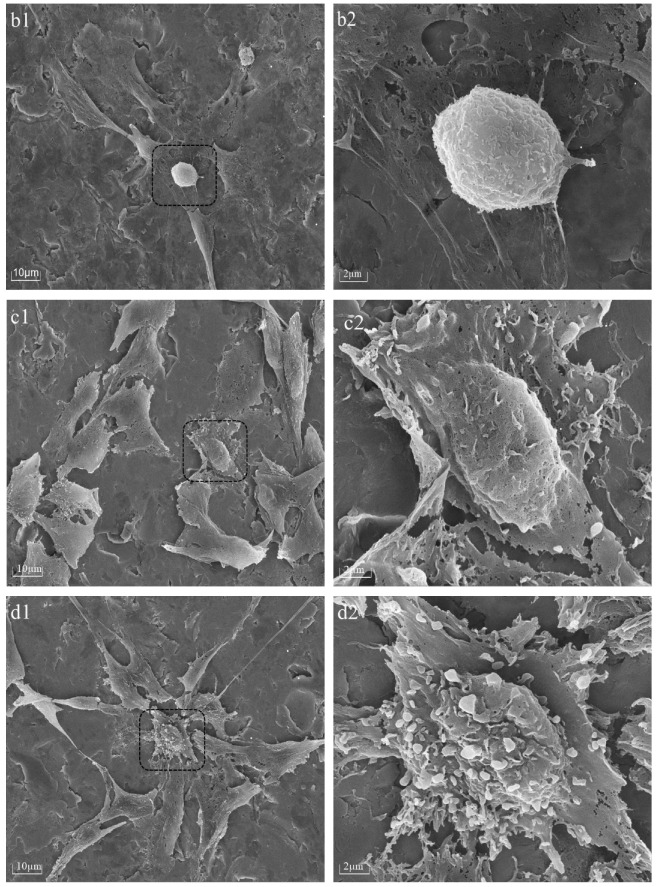

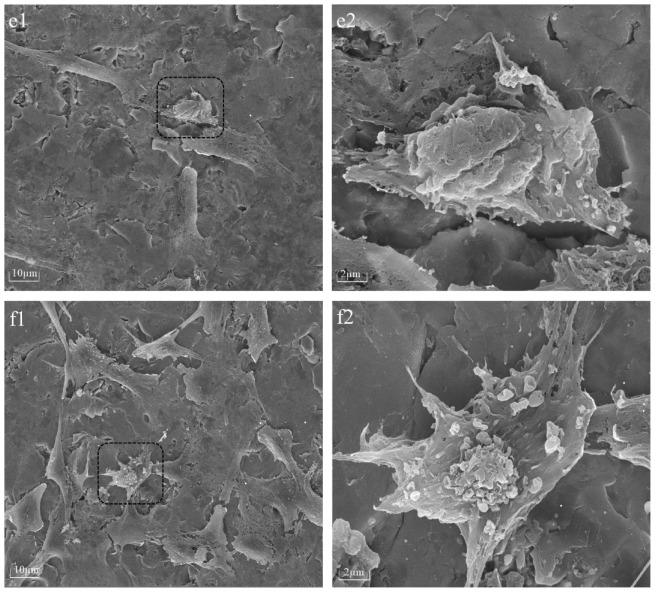
SEM images showed BMSC adhesion on the surface after 6 h of co-culture at 1000× (**a1**,**b1**,**c1**,**d1**) and 5000× magnification of the region in the dotted line square (**a2**,**b2**,**c2**,**d2**). (**a1**,**a2**) Deposition time of 0 s; (**b1**,**b2**) deposition time of 30 s; (**c1**,**c2**) deposition time of 45 s; (**d1**,**d2**) deposition time of 60 s; (**e1**,**e2**) deposition time of 90 s; (**f1**,**f2**) deposition time of 120 s. Cells adhered to the Ti6Al4V alloy surface and exhibited the formation of filopodia structures, promoting enhanced cell-to-cell interaction.

After 6 h of culture, a number of BMSCs were observed to have adhered to Ti6Al4V alloy surfaces with or without PPHA (Figure 5). With the increase in deposition time, different adhesion states of BMSCs on the surface were observed. As shown in Figure 5, more obvious cellular adhesion, cytoplasm protrusions, and extension were observed on the PPHA-Ti6Al4V alloy surface compared to the Ti6Al4V alloy. This observation demonstrates that the presence of PPHA on the surface of the Ti6Al4V alloy creates a favorable environment for the adhesion of BMSCs.

### 3.5. BMSCs Proliferation on PPHA-Ti6Al4V Alloy

**Figure 6:**
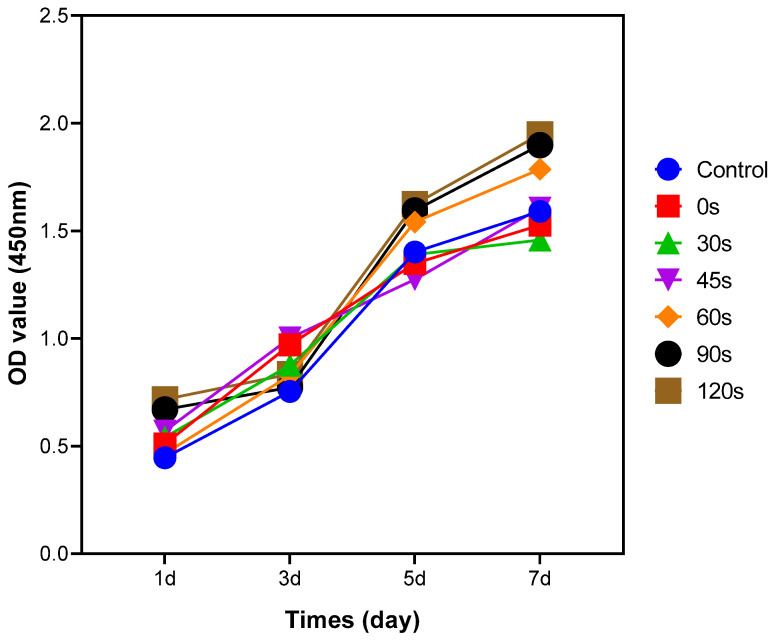
The results of CCK8 assay of BMSCs on PPHA-Ti6Al4V alloy at different deposition times: 0 s, 30 s, 45 s, 60 s, 90 s, 120 s. The OD values increased with BMSCs culture time.

CCK8 assays were carried out to analyze the effect of PPHA on BMSCs proliferation. After 1, 3, 5, and 7 days of cultivation with PPHA-Ti6Al4V alloy, the highest amount of cell proliferation was observed on the 7th day (Figure 6). Single-day observations revealed that the proliferation of BMSCs for PPHA deposition times of 60 s, 90 s, and 120 s exhibited a significant increase compared to deposition times of 0 s, 30 s, and 45 s (*p* < 0.05). However, two-way ANOVA indicated no significant difference between deposition times of 60 s, 90 s, and 120 s (*p* < 0.05). These findings suggest that PPHA on the surface of Ti6Al4V alloy can enhance BMSCs proliferation; this effect is partially dependent on the deposition time.

## 4. Discussion

The utilization of low-temperature atmospheric pressure plasma polymerization of n-heptylamine on the surface of Ti6Al4V alloy led to alterations in surface morphology, the introduction of an amino group on the surface, and an improvement in surface hydrophilicity. Consequently, these alterations facilitated the adhesion and proliferation of BMSCs. These effects were time-dependent when the deposition time of PPHA was less than 60 s. However, for longer deposition times, the effects did not exhibit a significant improvement compared to those observed exceeding a 60 s deposition time.

In several excellent reviews, plasma deposition mechanisms have been described in greater detail [18,19,20]. Within the plasma deposit, a limited number of structural components originating from the monomer and its chain-growth may be present [21]. Plasma polymer properties and structures might be further improved by a low-pressure regime and a short deposition time to solve its inherent stochastic and high excess energy characteristics [21]. Therefore, we developed an atmospheric pressure plasma system and conducted a thorough investigation into the impact of various deposition times. Hydrophilicity and surface energy occurred after storage in ambient air or water; these are referred to as post-plasma processes, which influence the hydrophilicity and surface energy by changing the surface chemical compositions [22]. Consequently, to eliminate post-plasma processes as much as possible in the current study, samples were preserved within airtight vacuum bags before undergoing surface analysis and cell cultivation.

Plasma polymer films commonly demonstrate resilient mechanical integrity, which can be attributed to their branched and substantial cross-linking in comparison to conventional polymers, which are composed of repeating units [22]. However, upon extending the deposition time to 90 s and 120 s, nanosized cavities (white region in Figure 2e,f) were observed within thin PPHA film layers in this current study, meaning a portion of the polymer was stripped away during the process. The occurrence of plasma etching could potentially result in the removal of atomic or molecular species from the surface of ultrathin PPHA polymer films on the material [23]. Ultrathin polymer films and coatings with deliberately integrated nanocavities have attracted significant attention due to their potential applications in corrosion prevention, separation processes, biosensing, release systems, etc. [24]. In this study, the formation of these nanocavities became increasingly obvious over an extended deposition time. These films hold significant potential for various applications, including delivery, storage, and release systems, as well as catalysis. Given the controllability of surface topography through plasma deposition time and power of the glow discharge [8,18], it is imperative to investigate appropriate plasma deposition conditions for the production of plasma polymer thin films with or without regulated free-volume cavities.

The XPS survey scans revealed the existence of nitrogen, carbon, and oxygen in PPHA across all deposition times during the surface elemental analysis (Table 1). The percentage of oxygen (O%) for the Ti6Al4V surface without PPHA was 22.52%. A passive oxide film that formed naturally on the Ti6Al4V alloy surface was found to be responsible for the significant corrosion resistance and bioactivity of the alloy [25]. This native passive film was thin and not completely stable [25]. During the low-temperature atmospheric pressure plasma polymerization, heptylamine was ionized to a high-energy excited state and then bonded to the surface in the form of amino groups (-NH_2_ and C-NH_2_) [26]. This phenomenon resulted in a high ratio of nitrogen to carbon (N/C), then decreased Ti6Al4V surface carbon contaminations, leading to surface activation. As presented in Figure 3 and Table 1, the results of N1s peak separation showed that the amount of -NH_2_ and C-NH_2_ significantly increased after plasma polymerization. In the present study, the nitrogen-to-carbon ratio (N/C) ranged from 6.34% to 8.38% at a discharge power of 9 W, in accordance with the findings of Kirby et al., who reported an N/C ratio between 5% and 7% at discharge powers ranging from 5 W to 20 W [9]. The NH_2_/C% ranged from 3.39% to 7.65%, aligning with prior research that reported NH_2_/C% within the range from 2% to 4% [4], but notably higher compared to the range from 0.96% to 1.37% in Kirby et al.’s study [9]. The heptylamine plasma exhibits a negative relationship between glow discharge power and nitrogen atom concentration [8,26]. High discharge power (e.g., 50 W) could produce less extent of abstraction of amine groups [26]. Therefore, the application of a low discharge power (9 W) in the current study might demonstrate a similarity to the theoretical elemental composition of heptylamine [26].

The hydrophilicity of plasma-modified materials is determined by the discharge power and polarity of the surface functional groups [4]. The surface exhibited increased hydrophilicity as the contact angle decreased, while the surface energy heightened as a result. At a PPHA deposition time of 30 s, the contact angle became 62.1° ± 1.6°, indicating that a large number of hydrophilic groups (amino groups) appeared on the Ti6A4V surface. However, when the deposition time extended to 90 s and 120 s, the water contact angle recovered, becoming similar to that of the Ti6Al4V alloy surface without PPHA. As reported, the water contact angle increased from 20° ± 1° to 125° ± 3° after heptylamine plasma polymerization onto porous alumina [27]. The observed increase in the water contact angle might be attributed to the dense, extensively interconnected, and heterogeneous structures of plasma polymer, which exhibited significant similarities to the monomer heptylamine itself [19]. The polymerization process exhibited competition between the formation of film-forming species through deposition and the ablation of the polymer film [21]. As the duration of the deposition process increased, a greater degree of polymer ablation was observed, resulting in the formation of nanocavities as observed during SEM. However, the presence of these nanocavities failed to enhance the material’s hydrophilic properties. In conclusion, the current study did not find that prolonging the PPHA deposition time would improve the material’s hydrophilicity.

This study revealed that the presence of PPHA on the surface of Ti6Al4V alloy resulted in increased proliferation of BMSCs, as determined via the CCK8 assay. This effect was observed at deposition times of 60 s, 90 s, or 120 s after a 7-day culture period. The cell activity of any substance is impacted by protein adsorption onto the surface. The heptylamine plasma polymer’s cross-linked structure [22] offers a notable advantage, as it greatly reduces the likelihood of protein diffusion into the plasma polymer. Consequently, the alterations in cell behavior could be exclusively attributed to surface interactions. The enhanced proliferation can be attributed to three factors: (1) the introduction of nano-scale surface morphology by PPHA, (2) modifications in the wettability of Ti6Al4V alloy surfaces, and (3) changes in the chemical components and functional groups present on the Ti6Al4V alloy surface. For the surface morphology, the surface nano topography was expanded by the PPHA film, increasing the number of available attachment sites for pseudo-like filopodia on extracellular membranes [28]. Moreover, the alteration of the surface morphology at the nano-scale level exerted a substantial impact on the material’s surface free energy and wettability [28]. When biological tissues come into contact, water molecules are the first entities to reach the surface. It is expected that the initial surface wettability plays a crucial role in influencing both protein adsorption onto the surface and cell adhesion. Consequently, the alteration of the material’s hydrophilicity augmented cellular activity. Water contact angles between 45° and 68° seem to be optimal for cell responses [29]. However, the results of our study revealed discrepancies in these findings. The proliferation of BMSCs was significantly enhanced when the deposition time was increased to 60 s, 90 s, and 120 s, accompanied by water contact angles ranging from 88° to 90°. This outcome indicated a non-linear relationship between the water contact angle/hydrophilicity and cell behavior [28]. The presence of synergistic effects resulted from the combination of nano-scaled topographical features and hydrophilicity at the interface between the substrate and cell [30].

The cell behavior of this study also suggested that the adhesion and proliferation of BMSCs were primarily influenced by the chemical composition, functional groups, and surface topography rather than the wettability [28]. During heptylamine plasma polymerization, the C−N bond undergoes cleavage, resulting in the generation of an •NH_2_ aminyl radical, which subsequently bonds with the Ti6Al4V surface. Amine-based surfaces have been documented as favorable substrates for cell cultivation, with the functional amino group associating with cell adhesion [31]. The adhesive glycoprotein fibronectin, an important extracellular matrix protein, has substantial involvement in crucial physiological mechanisms, mediating cell–cell adhesion and enhancing cell–substrate anchoring and spreading [32]. The fibronectin adsorption force obtained during our study clearly indicates a significant dependence on the chemical composition, with -NH_2_ > -CH_3_ >> -OH [33]. The synergistic effects of fibronectin adsorption force, organization, and cell traction force facilitated the rearrangement of adsorbed fibronectin molecules according to the cellular orientation, ultimately enhancing cell adhesion [33]. This enhancement was likely non-specific, potentially resulting from electrostatic interactions between the cells and amine groups present on the surface of the material [16]. This phenomenon of PPHA enhancement cell adhesion and proliferation has been observed not only in mesenchymal stem cells [26] but also in osteoblasts [15,34] and Schwann cells [35]. For cell proliferation, the amine concentration of amino groups arranged from 7% to 10% was promising from a tissue engineering perspective [36]. Notably, NH_2_ groups have been suggested to be permissive for the differentiation of mesenchymal stem cells into specialized cell types [37].

The operating parameters for the plasma polymerization study included the deposition time, the power of the glow discharge, the distance between the electrodes, and the monomer pressure in the reactor chamber [8]. The scope of this study was limited to investigating the influence of deposition time. Our findings have significant practical implications for the advancement of rapid, efficient, and cost-effective methods of surface functionalization in the field of tissue engineering. During polymerization, a competitive dynamic existed between the deposition of monomer species and their ablation [21]. In our investigation, it was observed that an increase in discharge power resulted in a higher occurrence of plasma etching on the surface of the Ti6Al4V alloy rather than the deposition of a polymer film. In the future, more studies are needed to polymerize uniform PPHA polymer and increase bonding energy, surface roughness, friction coefficient, and annealing in improving the aging resistance of PPHA polymeric coatings.

## 5. Conclusions

In this study, the surface modification of the Ti6Al4V alloy was successfully achieved by employing a custom-made low-temperature atmospheric pressure plasma polymerization system with n-heptylamine. The introduction of amino groups on the surface of Ti6Al4V alloy improved biocompatibility, and the modification effect was observed to be partially time-dependent. Within the scope of this study, a PPHA deposition time of 60 s was the most promising, as it not only offered a shorter duration but also facilitated the adhesion and proliferation of BMSCs.

## 6. Patents

There is one patent resulting from the work reported in this manuscript: A device for metal surface modification. China patent. 202120353356.9; 2021.

## Figures and Tables

**Table 1:** Relative chemical composition and atomic ratio determined via XPS for various deposition times. The atomic ratio of N/C and NH_2_/C on the PPHA-Ti6Al4V alloy surfaces was the most notably affected.

	Chemical Composition (%)			Atomic Ratio%		
Groups	C	O	N	N/C	NH_2_/C	NH_2_/N
0 s	74.02	22.52	3.46	4.68	3.39	72.48
30 s	80.24	14.67	5.09	6.34	5.14	81.03
45 s	81.73	11.65	6.62	8.10	6.71	82.83
60 s	87.02	6.86	6.12	7.03	6.72	95.54
90 s	85.84	7.17	6.99	8.14	7.31	89.84
120 s	83.02	10.02	6.96	8.38	7.65	91.28

## Data Availability

The data are contained within this article.
